# Chemistry and Therapeutics—Their Relation to Dentistry

**Published:** 1906-07

**Authors:** W. B. Fulton

**Affiliations:** Birmingham.


### CHEMISTRY AND THERAPEUTICS—THEIR RELATION TO DENTISTRY.

By W. B. FULTON, B. S., D. D. S. M. D., Birmingham.

(Alabama Dental Association.)

To discuss a theme so old as chemistry, and yet one so new as yesterday to the great masses of the human family, and not a very familiar subject to the dental fraternity, I must remark, is a pleasant task, but by no means an easy one. Why? Because every ninety-ninth student whom you meet wishes to know what chemistry has to do with dentistry; and what is true with the student is equally true of the practitioner, because he should be, if he is not, a student self-taught.

What relationship has chemistry to dentistry? Has it any?

Why, the whole of God's creation—pathology, anatomy, physiology, materia medica—in fact, dental science is built on chemic laws. Man is an embodiment of chemical elements—a fact recognized by Shakespeare when he caused the lips of Hamlet to say:

"Imperial Cæsar, dead and turned to clay,
Might stop a hole to keep the wind away.
O, that that earth which kept the world in awe
Should patch a wall to expel the winter's flaw!"

You plant a shrub, supply it with sodium, potassium, nitrogen, oxygen, and hydrogen, and there blooms forth an exquisitely beautiful flower—a rose—the decomposing molecules of which, floating on the atmosphere, come in contact with the Schneiderian membrane and produce a pleasant sensation in the brain, called a "sweet odor," or "fragrance," at the same time causing a series of molecular changes in the substance of the brain.

Without the science of chemistry, materia medica would have no place in science; it could not be. All drugs are compounded from chemical elements by chemical and physical laws. Take, for illustration, the alkaloids of plants, which are carbon compounds, acting as bases, combine with acid, and the result is a salt—for instance, muriate of cocaine, sulphate of morphine, and a great many others. Then follow chemical incompatibilities or counteracting agencies.

I once heard a physician (and he is considered one of the best surgeons in the South) remark that "a medical student does not need to know any chemistry." "If he does not," I said, "God pity the people where he begins to practice," for it would be practicing from a purely experimental standpoint. The only difference between the medical practitioner and him of dentistry is that of limitation. The dentist is a great deal less liable to do harm through his ignorance, only because he has a lesser chance.

Why not place iodine in a root canal to remove a broken broach or drill? Why, because iodine and iron form the insoluble iodine of iron which, penetrating and permeating the dentinal tubules, permanently stains the tooth structure. Why is hydrated sesquioxide of iron used to counteract the effect of arsenic on the gum tissue? How many ever thought of the answer? Here again there is a chemical reaction taking place between the iron and the arsenic, by which the inert arsenic of iron is formed.

I here call to memory a case when I was a college student. I had been told that nitrate of silver was a good thing to use to produce healthy granulation, and I proceeded to use it on this case where an upper third molar had been extracted several days previous. I rubbed it on, and, not having any evidence that the silver was dissolving, I continued to rub; and when it did make an appearance, I had an area an inch and a half square of a beautiful deep white eschar; and my patient began to use language foreign to my taste, words that would make Webster restless in his grave. I proceeded, and that in double-quick time, to a drug store and asked the druggist for something to counteract the effect, or at least to stop the effect of the silver nitrate; and he began by: "Ah—ah, let me see—." I cut him short by asking for a handful of sodium chloride—common table salt, as you all know. Making a strong aqueous solution and having my patient to rinse out the mouth, he was soon talking sense. Why? The sodium chloride combined with the silver nitrate and formed the inert silver chloride. I might thus continue on through the whole of materia medica and show one example after another, but it would make my paper too long.

One reason why the relationship of chemistry to dentistry is questioned is because we are not shown by our teachers the how, the when, and the where of these most interesting facts. Many of the teachers of chemistry in professional schools are doctors of medicine. We—and by "we" I mean our profession—have no text-book on chemistry. Another reason that its relationship is not recognized is a lack of fundamental training before entering a professional school. How can any one be interested in the chemistry of the action of hydrogen dioxide, or sodium dioxide, or any other dioxide, if he has no conception of oxygen, hydrogen, etc., and the laws governing them? They do not even awaken a curiosity in his brain. The truth of the whole matter is that he is willing to accept the thoughts of others and do things empirically.

When we come to consider physiology, we are ushered into a perfectly equipped chemical laboratory, upon which all structures and fluids of the body are dependent—a laboratory in which all the materials entering the body are prepared or digested for use by these structures.

Why is the saliva alkaline, and why is it important to keep it so? Because ptyalin acts only in an alkaline medium, and an acid medium destroys its function. On the other hand, pepsin requires an acid medium, and its function is destroyed by that of an alkaline, and so on. It is important that the dentist know these things, because a disturbance in the function of digestion produces an abnormal condition in the mouth and, indirectly, the teeth.

The theory of pyorrhea alveolaris is based upon one of chemistry, let it be that of an accumulation of uric acid, which I do not believe or that of an accumulation of calcific salts in the dentinal tubuli, which I consider more reasonable, when it is no longer necessary to maintain a physiological balance.

If we turn our attention to pathology, we are introduced to Miller through his theory that teeth decay because there are chemical decompositions taking place in the food on and between the teeth, furnishing a soil for fungi, which, in turn, produce lactic acid, and through its agency the teeth are destroyed. If we investigate Gray, one of the first questions asked is: What is the chemical composition of bone?

Turn to your metallurgy, without which few, if any, filling materials could be prepared, and you find it chemistry through and through. We live and breathe and have our being by permission from the laws of chemistry, and yet its importance or relation to dentistry has been questioned.

